# Design, Synthesis, and Activity Evaluation of Novel *N*-benzyl Deoxynojirimycin Derivatives for Use as α-Glucosidase Inhibitors

**DOI:** 10.3390/molecules24183309

**Published:** 2019-09-11

**Authors:** Fanxin Zeng, Zhongping Yin, Jiguang Chen, Xuliang Nie, Ping Lin, Tao Lu, Meng Wang, Dayong Peng

**Affiliations:** 1 Jiangxi Key Laboratory of Natural Products and Functional Food, Jiangxi Agricultural University, Nanchang 330045, China; 2 College of Food Science and Engineering, Jiangxi Agricultural University, Nanchang 330045, China; 3 Jiangxi Academy of Forestry, Nanchang 330013, China; 4 Collaborative Innovation Center of Jiangxi Typical, Trees Cultivation and Utilization, Nanchang 330045, China

**Keywords:** *N*-benzyl deoxynojirimycin derivatives, α-glucosidase inhibitor, structure-activity analysis, molecular docking

## Abstract

To obtain α-glucosidase inhibitors with high activity, 19 NB-DNJDs (*N*-benzyl-deoxynojirimycin derivatives) were designed and synthesized. The results indicated that the 19 NB-DNJDs displayed different inhibitory activities towards α-glucosidase in vitro. Compound **18a** (1-(4-hydroxy-3-methoxybenzyl)-2-(hydroxymethyl) piperidine-3,4,5-triol) showed the highest activity, with an IC_50_ value of 0.207 ± 0.11 mM, followed by **18b** (1-(3-bromo-4-hydroxy-5-methoxybenzyl)-2-(hydroxymethyl) piperidine-3,4,5-triol, IC_50_: 0.276 ± 0.13 mM). Both IC_50_ values of **18a** and **18b** were significantly lower than that of acarbose (IC_50_: 0.353 ± 0.09 mM). According to the structure-activity analysis, substitution of the benzyl and bromine groups on the benzene ring decreased the inhibition activity, while methoxy and hydroxyl group substitution increased the activity, especially with the hydroxyl group substitution. Molecular docking results showed that three hydrogen bonds were formed between compound **18a** and amino acids in the active site of α-glucosidase. Additionally, an arene–arene interaction was also modelled between the phenyl ring of compound **18a** and Arg 315. The three hydrogen bonds and the arene–arene interaction resulted in a low binding energy (−5.8 kcal/mol) and gave **18a** a higher inhibition activity. Consequently, compound **18a** is a promising candidate as a new α-glucosidase inhibitor for the treatment of type Ⅱ diabetes.

## 1. Introduction

Iminosugars are carbohydrate mimics in which the endocyclic oxygen has been replaced with a nitrogen atom. Some iminosugars are regarded as potential inhibitors of glycosidase. It has been reported that the bioactivity of iminosugars is related to their ability to mimic the transition state of pyranosidic or furanosidic units of the natural substrate during glycosidic bond cleavage [1,2].

The first generation of iminosugars came from the discovery of nojirimycin (NJ) and its biological activity on both α- and β-glucosidase. However, the presence of a hydroxyl group at C-1 results in the instability of NJ [1]. Therefore, a more stable glucosidase inhibitor named 1-deoxynojirimycin(1-DNJ) was synthesized via the reduction of nojirimycin. Unfortunately, even though 1-DNJ has excellent inhibitory ability in vitro, its in vivo activity has not proven to be as good as that in vitro [3]. Nakagawa and co-workers evaluated the metabolic fate of orally ingested 1-DNJ extracted from mulberry and found that bacterial digestion of 1-DNJ in the gastrointestinal system may occur before its absorption, as they did not detect any 1-DNJ metabolites in the plasma [4]. According to Su et al, 1-DNJ was quickly metabolized and excreted, and it therefore had no significant hypoglycaemic effect in vivo [5]. Faber et al. studied the pharmacokinetics of *N*-methyl-DNJ, as it has a molecular structure similar to that of 1-DNJ, in rats after intravenous administration. They found that the compound exhibited a rapid biphasic reduction in plasma, with an initial *t*_1/2_ of 4.5 min and a terminal *t*_1/2_ of 32 min. At 120 min after administration, 80% of the dose was recovered from the urine and was unchanged [6]. In view of NJ’s instability and 1-DNJ’s unsatisfactory inhibition of α-glucosidase in vivo, researchers began investigating other inhibitors derived from 1-DNJ. The approval of Glyset (miglitol) for the treatment of complications associated with type II diabetes indicated promising prospects for the application of this type of compound.

In recent decades, many investigations on the synthesis, characterization and biological activities of *N*-substituted 1-DNJ have been performed, but most of these studies were conducted to find inhibitors of ceramide glucosyltransferase (CGT), used to treat Gaucher’s disease [7,8,9,10,11,12,13]. For example, *N*-alkyl- and *N*-alkenyl-DNJ derivatives inhibit CGT, and it was found that those derivatives with longer chains were better CGT inhibitors. Adamantan-1-yl-methoxy-functionalized 1-DNJ derivatives are more potent inhibitors of glucosylceramide synthase than the therapeutic agent Zavesca that is indicated for the treatment of Gaucher disease [13]. *N*-pentafluorobenzyl-1-DNJ was synthesized, and it was found that it inhibited the secretion of interleukin-4 and the expression of CD4. This molecule may be a new immunosuppressant candidate for treating Th2-mediated immune diseases [14]. In other literature, *N*-Alkyl-DNJ also inhibits α-glucosidase, and the position of the alkyl chain (α-1-C, β-1-C, or *N*-alkyl derivatives) greatly influences the selectivity and potency [15]. Hybrids of 1-DNJ and 5-aryl-1,2,3-triazole were synthesised and were found to be more potent inhibitors of angiogenesis in vitro than DNJ or 5-aryl-1,2,3-triazole alone [16]. Few studies have been carried out with the aim of developing α-glucosidase inhibitors from the series of N-benzyl-DNJ derivatives (NB-DNJDs). In the present paper, several NB-DNJDs were synthesized and evaluated for α-glucosidase inhibition. Furthermore, molecular docking was performed to investigate the interaction between the novel inhibitors and α-glucosidase, and the results were further used to interpret the inhibition mechanism.

## 2. Results and Discussion

### 2.1. Chemistry

#### 2.1.1. 1-DNJ Preparation

**Scheme 1:**
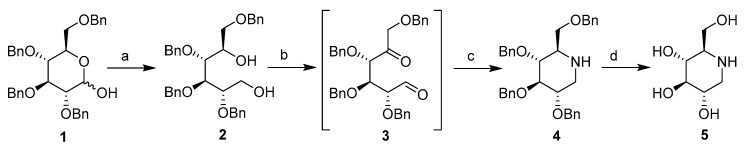
1-DNJ synthesis. Reagents and conditions: (**a**) LiAlH_4_, THF, 0 °C; (**b**) (COCl)_2_, DMSO, DCM; (**c**) NaBH_3_CN, CH_3_COONH_4_, Na_2_SO_4_, MeOH; and (**d**) H_2_, Pd/C, r.t.

1-DNJ were synthesized according to the methods of Wennekes and Matos with slight modification (shown as Scheme 1) using 2,3,4,5-tetra-O-benzyl-d-glucopyranose (compound **1** in Scheme 1) as the starting compound [13,17,18].

2,3,4,5-tetra-O-benzyl-d-glucopyranose **1**, through a successive hemiacetal reduction by lithium aluminium hydride, gave the corresponding alcohol **2** in the form of a colourless viscous syrup with a quantitative yield. A double Pfitzner‒Moffatt oxidation of the 1,5-diol **2** was carried out at −78 °C. This step initially involved the utilization of DMSO activated by treatment with oxalyl chloride in CH_2_Cl_2_. The reaction was terminated after 2 h by the addition of triethylamine. The reduced pressure evaporation of solvents furnished the unstable 1,5-dicarbonyl sugar derivative **3** that was used in the next step without purification. Then, the crude 1,5-dicarbonyl sugar derivative **3** was cyclized by double reductive amination using ammonium formate as a source of nitrogen and NaBH_3_CN as the source of hydrogen. These conditions could reproducibly generate compound **4** in yields of 60–65% after the first three steps. The amine **4** was exposed to Pd/C-catalysed hydrogenolysis at 5 bars in an ethanol/hydrochloric acid mixture to cause full deprotection and yield lead compound **5**.

#### 2.1.2. Synthesis of Vanillin Derivatives

**Scheme 2:**
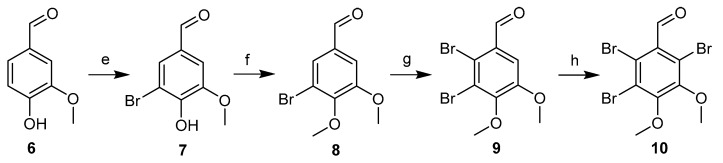
Synthesis of the intermediate compounds **7**–**10**. Reagents and conditions:(e) HAc, Br_2_; (f) MeI, K_2_CO_3_; (g) HAc, Br_2_; and (h) NBS, H_2_SO_4_.

The synthetic routes to the intermediate products **6**–**10** are shown in Scheme 2. First, commercially available vanillin **6** was reacted with bromine in CH_3_OH at 0 °C to produce compound **7** with a 95% yield. Compound **7** was methylated with CH_3_I in DMF to provide compound **8** with a 97% yield. Then, a second bromination of compound **8** was performed using double the amount of equivalent bromine in acetic acid at 60 °C, yielding compound **9** (60% yield). This compound was further brominated with *N*-bromosuccinimide (NBS) in concentrated sulfuric acid to generate compound **10** (48% yield) [19,20].

#### 2.1.3. Synthesis of NB-DNJDs

**Scheme 3:**
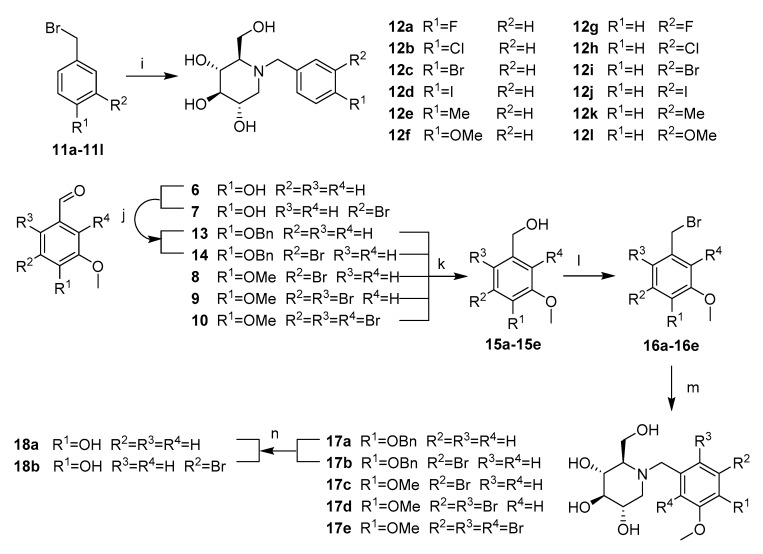
Synthesis of NB-DNJDs **12a**–**12l**, **17a**–**17e** and **18a**–**18b**. Reagents and conditions:(i) K_2_CO_3_, DMF, 1-DNJ, 80 °C; (j) BnCl, K_2_CO_3_, DMF, 80 °C; (k) NaBH_4_, DMF, 0 °C; (l) BBr_3_,DCM; (m) K_2_CO_3_, DMF, 1-DNJ, 80 °C; and (n) H_2_, Pd/C, r.t.

Compounds **12a**–**12l** were synthesized by adapting the nucleophilic substitution reaction shown in Scheme 3. Condensation of the starting compound 1-DNJ with commercially available benzyl bromide derivatives **11a**–**11b** in the presence of potassium carbonate afforded the corresponding *N*-benzyl deoxynojirimycin derivatives **12a**–**12l** in high yields.

The synthetic routes to targets **17a**–**17e** and **18a**–**18b** shown in Scheme 3 commenced with the intermediate products **6**–**10**. First, in the presence of potassium carbonate, the hydroxyl groups of compounds **6** and **7** were reacted with benzyl bromide in methanol to produce the benzyl protected aldehyde in **13** and **14** in quantitative yield [21]. Then, a successive aldehyde reduction/bromination/nucleophilic substitution of compounds **8**–**10** and **13**–**14** produced targets **17a**–**17e** [11,22,23]. Finally, the debenzylation of targets **17a**–**17b** was achieved using palladium on carbon in methanol/ethyl acetate (*v:v* = 1:1) to yield the desired targets **18a**–**18b** [24].

### 2.2. Inhibition of α-Glucosidase by NB-DNJDs

α-Glucosidase (EC 3.2.1.20) is a hydrolase enzyme which actually destroys the disaccharides and oligosaccharides into glucose. The hypoglycaemic agents slow down the digestion and assimilation of simple carbohydrates through α-glucosidase inhibition [25]. Miglitol, voglibose, and acarbose are well-known α-glucosidase inhibitors used to suppress hyperglycaemia. α-glucosidase has been investigated as a potential target enzyme in the treatment of type 2 diabetes.

The inhibitory activities of these 19 newly synthesized NB-DNJDs with aryl functionalities (**12a**–**12l**, **17a**–**17e** and **18a**–**18b**, with the purity range from 92~98%) towards α-glucosidase were evaluated by an in vitro microreaction model in 96-well plates using acarbose as the positive control. The results indicated that the 19 NB-DNJDs displayed different inhibitory activities (Table 1). Among the 19 compounds tested, **18a** showed the highest activity, with an IC_50_ value of 0.207 ± 0.11 mM, followed by **18b** (IC_50_: 0.276 ± 0.13 mM). Both IC_50_ values of **18a** and **18b** were significantly lower than that of acarbose (IC_50_: 0.353 ± 0.09 mM).

The inhibitory activities of **12f** and **12l** were much stronger than those of **12a**–**12e** and **12g**–**12k**, suggesting that the introduction of a methoxy group into the phenyl ring of NB-DNJDs might be a better approach to improving the activity rather than the introduction of halogen atoms or methyl groups. 

Compounds **17a**–**17e** are a family of compounds with the same parent structure, but they showed different inhibition activities towards α-glucosidase (Table 1). Among the 5 compounds, **17c** (3-Br, 4,5-di-OMe) showed the highest activity, followed by **17d** (2,3-Br_2_, 4,5-di-OMe) and **17e** (2,3,6-Br_3_, 4,5-di-OMe), while **17a** (1-OBn) and **17b** (1-OBn, 2-Br) had weak activities. These results indicated that the number of bromines in the benzene ring was critical and that having two methoxy groups in the benzene ring was important as well. 

There are many reports about introducing bromine atoms on the aromatic ring to search a potent α-glucosidase inhibitory. For example, Zawawi and his co-workers [26] found that the position of bromine atoms on the aromatic ring of the inhibitors affected the activity on α-glucosidase. It indicated that electron withdrawing moieties, like NO_2_, the Br and Cl, are more favourable for the interactions with active site residues; Kim et al. [27,28,29] reported that the number of Br atoms in a benzene ring may significantly influence the activity of inhibitors. They found that highly brominated bromophenols might mimic the spatial structure of α-glucosidase. In addition, highly brominated catechols are susceptible to oxidation and converted into o-quinones. They will strongly bind to the enzymes due to debrominated aromatic nucleophilic substitution. However, the results in our study indicated that bromine substitution may reduce the inhibition activity of NB-DNJDs. The lower the number of bromine substitutions on the benzene ring is, the higher the activity. However, methoxy and hydroxyl group substitutions increased the inhibition activity. The hydroxyl group substitution, in particular, led to a significant improvement in activity. The comparative analysis results of the activities and structures of compounds **17c**, **17d**, **18a** and **18b** indicated that NB-DNJDs with more hydroxyl groups and no bromine in the benzene ring may have a much higher α-glucosidase inhibition activity and therefore be worth further investigation for the treatment of diabetes.

### 2.3. Enzyme Kinetics Study

To study the mechanism of inhibition of these compounds towards α-glucosidase, kinetic studies of **18a** were performed, and the results were analysed using non-linear regression analysis. In the enzyme kinetics studies, the enzyme activity was measured at different times using different concentrations of the substrate p-nitrophenyl α-d-glucopyranoside (0.1, 0.2, 0.8, 1.0, 1.2, 1.6, 1.8, 2.0, 2.5, 5 mM). The kinetics of the enzymatic reaction were fit to the Michaelis‒Menten equation. The Michaelis‒Menten equation is as follows:

*V* = *V*max[*S*]/(*K*m + [*S*]).

**Figure 1:**
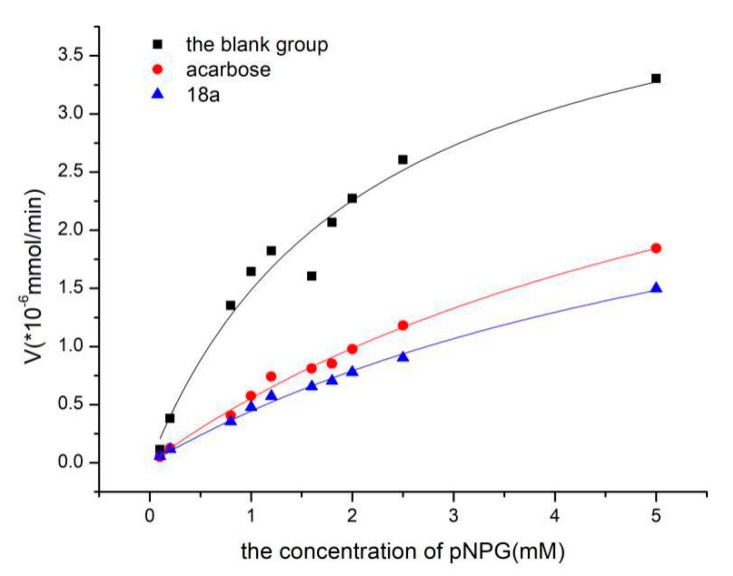
The inhibitory effects of acarbose and **18a** towards α-glucosidase analysed using non-linear regression analysis.

In this equation, [*S*] is the concentration of the substrate, *V*max is the maximum reaction velocity of the enzymatic reaction, and *K*m is the Michaelis constant. To determine the *K_i_* constants for compounds **18a** and **18b**, non-linear-regression plots were analysed (Figure 1). Non-linear fitting was performed with the help of Origin 8.5, which can calculate the *V*max and *K*m values. The *K_i_* constant was determined as follows:

*Ki* = [*I*]/(*K*’m/*K*m − 1).

In this formula, [*I*] is the concentration of inhibitor, *K*’m is the Michaelis constant of the experimental group, and *K*m is the Michaelis constant of the blank group.

The *K_i_* for compound **18a** was 0.110 mM, which was calculated according to the data in Table 2. The *K_i_* for acarbose was 0.113 mM. The results showed that compound **18a** was the most potent α-glucosidase inhibitor and therefore may be useful for treating type Ⅱ diabetes.

### 2.4. Molecular Docking Simulation of NB-DNJD and α-Glucosidase

**Figure 2:**
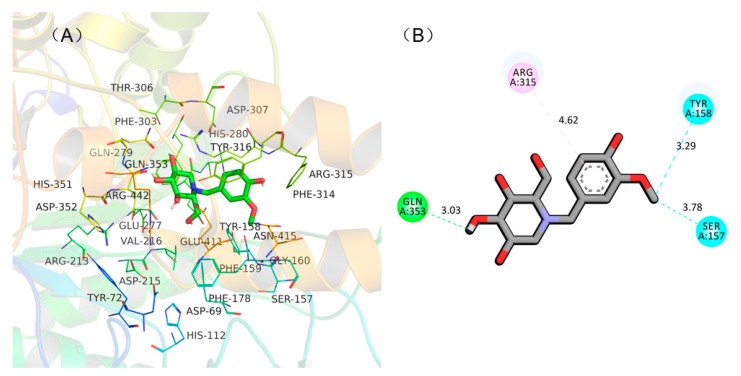
Docking conformation of compound **18a** in the active site of α-glucosidase. (**A**) Predicted docking pose of **18a** with α-glucosidase; (**B**) a plane figure of the interaction between **18a** and nearby residues, and hydrogen bonds are represented in green and blue lines, while π-π stacked is represented in purple line.

To understand the binding mode between NB-DNJD and α-glucosidase to assist in interpreting the mechanism of inhibition of NB-DNJD, a molecular docking simulation was carried out using AutoDock 4.2. All 19 of the NB-DNJDs were docked into the active site of α-glucosidase (PDB: 3A4A), and the hydrogen bonds, arene–arene interactions, and binding energy between NB-DNJDs and α-glucosidase were analysed. Among the 19 NB-DNJDs, compound **18a** displayed the highest inhibition activity and was therefore used as an example to illustrate why compounds with a similar molecule structure to **18a** had high activities. In the model, three hydrogen bonds (within 4 Å) formed between compound **18a** and two amino acids in the active site (shown in Figure 2). Compound **18a** bound with Tyr 158, Gln 353 and Ser 157 by hydrogen bond at distance of 3.29, 3.03, and 3.78 Å, respectively, and interacted with Arg 315 via π‒π interaction. These three hydrogen bonds and π‒π interaction between the enzyme and compound **18a** (inhibitor) resulted in a low binding energy (‒5.8 kcal/mol), which gave **18a** a strong inhibitory activity to α-glucosidase. 

## 3. Conclusions

To obtain high activity inhibitors of α-glucosidase, nineteen NB-DNJDs were designed and synthesised. The results indicated that the 19 NB-DNJDs displayed different inhibitory activities towards α-glucosidase in vitro. Compound **18a** showed the highest activity, with an IC_50_ of 0.207 ± 0.11 mM, followed by **18b** (IC_50_: 0.276 ± 0.13 mM). The IC_50_ values of both **18a** and **18b** were significantly lower than that of acarbose (IC_50_: 0.353 ± 0.09 mM). The results of molecular docking simulation indicated that three hydrogen bonds were formed between compound **18a** and amino acids in the active site of α-glucosidase, and an arene–arene interaction was also observed between the phenyl ring of compound **18a** and Arg 315. The three hydrogen bonds and the arene–arene interaction resulted in a low binding energy (−5.8 kcal/mol) and therefore led to a high inhibition activity of compound **18a**. This activity has a close relationship with the phenolic unit in the molecules. However, further activity evaluations of compound **18a** on other digestive enzymes, such as a-amylase, maltase-glucoamylase and β-glucosidase [30], are needed to develop its application value in the future.

## 4. Experimental Section

### 4.1. Chemistry 

All materials and reagents were purchased from commercial suppliers. Melting points were determined on electrothermal digital melting point apparatus and were uncorrected. TLC was performed on 0.20-mm Silica Gel 60 F_254_ plates (Qingdao Ocean Chemical Factory, Shandong, China). Nuclear magnetic resonance (NMR) spectra were recorded at 400 MHz on a Varian NMR spectrometer. The NMR data of the known compounds are consistent with previous reports.

#### 4.1.1. Synthesis of 1-(4-fluorobenzyl)-2-(hydroxymethyl) piperidine-3,4,5-triol (**12a**)

K_2_CO_3_ (4 mmol) was added to a solution of 1-DNJ (2 mmol) and 4-fluorobenzyl bromide (2.4 mmol) in DMF. The mixture was stirred at r.t. until 1-DNJ was depleted, as determined by TLC analysis. Then, the mixture was washed with EtOAc/H_2_O (*v/v* = 1:1). The aqueous phase was washed 3 times with EtOAc. The combined extracts were washed successively with 1 M aq HCl and saturated aq NaHCO_3_, dried over anhydrous Na_2_SO_4_, and evaporated by vacuum to obtain a residue. The residue was purified using silica gel column chromatography (dichloromethane: methyl alcohol = 15:1) to produce a yellow solid (83%). ^1^H NMR (400 MHz, DMSO-*d*_6_) δ 7.35 (dd, *J* = 14.3, 7.5 Hz, 1H), 7.16 (d, *J* = 6.0 Hz, 2H), 7.05 (t, *J* = 8.6 Hz, 1H), 4.78 (d, *J* = 5.3 Hz, 1H), 4.72 (d, *J* = 4.0 Hz, 1H), 4.62 (d, *J* = 4.3 Hz, 1H), 4.41 (s, 1H), 4.26 (d, *J* = 14.0 Hz, 1H), 3.94 (d, *J* = 11.6 Hz, 1H), 3.67–3.59 (m, 1H), 3.20–3.05 (m, 3H), 2.98–2.90 (m, 1H), 2.60 (dd, *J* = 11.1, 4.5 Hz, 1H), 2.03 (d, *J* = 8.4 Hz, 1H), 1.75 (t, *J* = 10.8 Hz, 1H). ^13^C NMR (100 MHz, DMSO-*d*_6_) δ 162.30, 159.89, 135.68, 130.51, 114.80, 114.59, 79.09, 70.91, 69.14, 67.86, 59.79, 56.62, 55.56; HRMS: C_13_H_18_FNO_4_ for +, calculated 271.1215, found 271.1289; [α]${}_{D}^{24}$ −0.206 (0.53, DMSO).

#### 4.1.2. Synthesis of 1-(4-chlorobenzyl)-2-(hydroxymethyl) piperidine-3,4,5-triol (**12b**)

Compound **12b** was prepared in a manner similar to the procedure described for the preparation of **12a** to yield a solid. The residue was purified using silica gel column chromatography (dichloromethane: methyl alcohol = 15:1) to produce a yellow solid (90%). ^1^H NMR (400 MHz, DMSO-*d*_6_) δ 7.41 (s, 1H), 7.34 (d, *J* = 7.6 Hz, 1H), 7.30 (d, *J* = 1.7 Hz, 2H), 4.84–4.68 (m, 2H), 4.63 (d, *J* = 3.8 Hz, 1H), 4.42 (s, 1H), 4.25 (d, *J* = 13.8 Hz, 1H), 3.94 (d, *J* = 11.3 Hz, 1H), 3.69–3.58 (m, 1H), 3.14 (d, *J* = 13.8 Hz, 2H), 3.11–3.05 (m, 1H), 2.99–2.91 (m, 1H), 2.58 (dd, *J* = 11.0, 4.4 Hz, 1H), 2.02 (d, *J* = 7.6 Hz, 1H), 1.76 (s, 1H); ^13^C NMR (100 MHz, DMSO-*d*_6_) δ 138.70, 134.95, 131.12, 130.51, 128.98, 127.98, 79.06, 70.89, 69.13, 67.85, 59.79, 56.72, 55.63; HRMS: C_13_H_18_ClNO_4_ for +, calculated 287.0919, found 271.1289; [α]${}_{D}^{23}$ −0.321 (0.96, DMSO).

#### 4.1.3. Synthesis of 1-(4-bromobenzyl)-2-(hydroxymethyl) Piperidine-3,4,5-triol (**12c**)

Compound **12c** was prepared in a manner similar to the procedure described for the preparation of **12a** to yield a solid. The residue was purified using silica gel column chromatography (dichloromethane: methyl alcohol = 20:1) to produce a yellow solid (78%). ^1^H NMR (400 MHz, DMSO-*d*_6_) δ 7.46 (s, 1H), 7.34 (d, *J* = 7.5 Hz, 1H), 7.24 (d, *J* = 7.3 Hz, 1H), 7.19 (t, *J* = 7.6 Hz, 1H), 4.69 (d, *J* = 5.3 Hz, 1H), 4.63 (d, *J* = 4.1 Hz, 1H), 4.54 (d, *J* = 4.4 Hz, 1H), 4.33 (t, *J* = 4.8 Hz, 1H), 4.16 (d, *J* = 13.9 Hz, 1H), 3.85 (d, *J* = 10.5 Hz, 1H), 3.58–3.51 (m, 1H), 3.10–2.95 (m, 3H), 2.85 (td, *J* = 8.7, 4.1 Hz, 1H), 2.49 (dd, *J* = 11.0, 4.5 Hz, 1H), 2.42 (s, 1H); ^13^C NMR (100 MHz, DMSO-*d*_6_) δ 139.06, 130.92, 119.65, 79.01, 70.83, 69.08, 67.86, 59.73, 56.68, 55.68; HRMS: C_13_H_18_BrNO_4_ for +, calculated 331.0414, found 331.0492; [α]${}_{D}^{23}$ −0.152 (0.65, DMSO).

#### 4.1.4. Synthesis of 2-(hydroxymethyl)-1-(4-iodobenzyl) Piperidine-3,4,5-triol (**12d**)

Compound **12d** was prepared in a manner similar to the procedure described for the preparation of **12a** to yield a solid. The residue was purified using silica gel column chromatography (dichloromethane: methyl alcohol = 15:1) to produce a yellow solid (86%). ^1^H NMR (400 MHz, DMSO-*d*_6_) δ 7.72 (s, 1H), 7.60 (d, *J* = 7.7 Hz, 1H), 7.34 (d, *J* = 7.6 Hz, 1H), 7.13 (t, *J* = 7.6 Hz, 1H), 4.77 (d, *J* = 5.5 Hz, 1H), 4.71 (d, *J* = 4.1 Hz, 1H), 4.63 (d, *J* = 4.4 Hz, 1H), 4.41 (t, *J* = 4.8 Hz, 1H), 4.22 (d, *J* = 13.8 Hz, 1H), 3.93 (d, *J* = 8.9 Hz, 1H), 3.67–3.59 (m, 1H), 3.10 (dd, *J* = 20.2, 9.5 Hz, 3H), 2.95 (dd, J = 8.6, 4.4 Hz, 1H), 2.58 (dd, *J* = 11.3, 4.6 Hz, 1H), 2.00 (d, *J* = 8.0 Hz, 1H), 1.73 (t, *J* = 10.6 Hz, 1H); ^13^C NMR (100 MHz, DMSO-*d*_6_) δ 139.59, 136.78, 131.14, 92.28, 79.04, 70.89, 69.13, 67.89, 59.81, 56.77, 55.85; HRMS: C_13_H_18_INO_4_ for +, calculated 379.0276, found 379.0249; [α]${}_{D}^{22}$ −0.144 (0.53, DMSO).

#### 4.1.5. Synthesis of 2-(hydroxymethyl)-1-(4-methylbenzyl) Piperidine-3,4,5-triol (**12e**)

Compound **12e** was prepared in a manner similar to the procedure described for the preparation of **12a** to yield a solid. The residue was purified using silica gel column chromatography (dichloromethane: methyl alcohol = 10:1) to produce a white solid (87%). ^1^H NMR (400 MHz, DMSO-*d*_6_) δ 7.19 (t, *J* = 7.5 Hz, 1H), 7.12 (s, 1H), 7.09 (d, *J* = 7.1 Hz, 1H), 7.04 (d, *J* = 7.4 Hz, 1H), 4.76 (d, *J* = 5.3 Hz, 1H), 4.70 (d, *J* = 4.0 Hz, 1H), 4.59 (d, *J* = 4.4 Hz, 1H), 4.33 (d, *J* = 5.1 Hz, 1H), 4.22 (d, *J* = 13.4 Hz, 1H), 3.95 (d, *J* = 10.6 Hz, 1H), 3.68–3.60 (m, 1H), 3.16–3.06 (m, 2H), 3.03 (d, *J* = 13.9 Hz, 1H), 2.93 (dd, *J* = 13.6, 9.3 Hz, 1H), 2.62 (dd, *J* = 11.1, 4.5 Hz, 1H), 2.30 (s, 3H), 1.98 (d, *J* = 8.1 Hz, 1H), 1.76 (s, 2H), 1.69 (t, *J* = 10.9 Hz, 1H); ^13^C NMR (100 MHz, DMSO-*d*_6_) δ 136.31, 135.59, 128.70, 79.13, 70.96, 69.17, 67.92, 59.76, 56.64, 56.23, 20.66; HRMS: C_14_H_21_NO_4_ for +, calculated 267.1466, found 267.1531; [α]${}_{D}^{24}$ −0.194 (0.63, DMSO).

#### 4.1.6. Synthesis of 2-(hydroxymethyl)-1-(4-methoxybenzyl) Piperidine-3,4,5-triol (**12f**)

Compound **12f** was prepared in a manner similar to the procedure described for the preparation of **12a** to yield a solid. The residue was purified using silica gel column chromatography (dichloromethane: methyl alcohol = 10:1) to produce a white solid (89%). ^1^H NMR (400 MHz, DMSO-*d*_6_) δ 7.35 (dd, *J* = 14.3, 7.5 Hz, 1H), 7.16 (d, *J* = 6.0 Hz, 2H), 7.05 (t, *J* = 8.6 Hz, 1H), 4.78 (d, *J* = 5.3 Hz, 1H), 4.72 (d, *J* = 4.0 Hz, 1H), 4.62 (d, *J* = 4.3 Hz, 1H), 4.41 (s, 1H), 4.26 (d, *J* = 14.0 Hz, 1H), 3.94 (d, *J* = 11.6 Hz, 1H), 3.67–3.59 (m, 1H), 3.20–3.05 (m, 3H), 2.98–2.90 (m, 1H), 2.60 (dd, *J* = 11.1, 4.5 Hz, 1H), 2.03 (d, *J* = 8.4 Hz, 1H), 1.75 (t, *J* = 10.8 Hz, 1H); ^13^C NMR (100 MHz, DMSO-*d*_6_) δ 158.30, 130.35, 113.52, 78.79, 70.51, 68.19, 62.89, 55.69, 55.06, 52.23, 7.35; HRMS: C_14_H_21_NO_5_ for +, calculated 283.1415, found 283.1495; [α]${}_{D}^{23}$ −0.142 (1.37, DMSO).

#### 4.1.7. Synthesis of 1-(3-fluorobenzyl)-2-(hydroxymethyl) Piperidine-3,4,5-triol (**12g**)

Compound **12g** was prepared in a manner similar to the procedure described for the preparation of **12a** to yield a solid. The residue was purified using silica gel column chromatography (dichloromethane: methyl alcohol = 10:1) to produce a yellow solid (91%). ^1^H NMR (400 MHz, DMSO-*d*_6_) δ 7.38–7.31 (m, 2H), 7.13 (t, *J* = 8.6 Hz, 2H), 4.77 (d, *J* = 5.5 Hz, 1H), 4.71 (d, *J* = 4.1 Hz, 1H), 4.60 (d, *J* = 4.5 Hz, 1H), 4.38 (t, *J* = 5.0 Hz, 1H), 4.21 (d, *J* = 13.6 Hz, 1H), 3.95 (d, *J* = 11.2 Hz, 1H), 3.68–3.60 (m, 1H), 3.17–3.04 (m, 3H), 2.93 (dt, *J* = 12.7, 6.3 Hz, 1H), 2.58 (dd, *J* = 11.4, 4.5 Hz, 1H), 2.00 (d, *J* = 8.9 Hz, 1H), 1.71 (t, *J* = 10.7 Hz, 1H); ^13^C NMR (100 MHz, DMSO-*d*_6_) δ 163.43, 161.01, 142.91, 129.87, 124.61, 115.16, 114.95, 113.46, 113.25, 79.03, 70.88, 69.11, 67.85, 59.82, 56.85, 55.84; HRMS: C_13_H_18_FNO_4_ for +, calculated 271.1215, found 271.1289; [α]${}_{D}^{24}$ −0.019 (0.19, DMSO).

#### 4.1.8. Synthesis of 1-(3-chlorobenzyl)-2-(hydroxymethyl) Piperidine-3,4,5-triol (**12h**)

Compound **12h** was prepared in a manner similar to the procedure described for the preparation of **12a** to yield a solid. The residue was purified using silica gel column chromatography (dichloromethane: methyl alcohol = 15:1) to produce a yellow solid (79%). ^1^H NMR (400 MHz, DMSO-*d*_6_) δ 7.36 (s, 4H), 4.74 (d, *J* = 22.6 Hz, 2H), 4.60 (s, 1H), 4.40 (s, 1H), 4.23 (d, *J* = 13.9 Hz, 1H), 3.94 (d, *J* = 13.5 Hz, 1H), 3.64 (s, 1H), 3.12 (d, *J* = 14.4 Hz, 3H), 2.94 (s, 1H), 2.90 (s, 1H), 2.74 (s, 1H), 2.57 (d, *J* = 11.1 Hz, 1H), 2.02 (s, 1H), 1.79–1.68 (m, 1H); ^13^C NMR (100 MHz, DMSO-*d*_6_) δ 142.42, 132.88, 129.87, 128.30, 127.34, 126.60, 79.03, 70.84, 69.11, 67.77, 59.69, 56.77, 55.74; HRMS: C_13_H_18_ClNO_4_ for +, calculated 287.0919, found 287.0983; [α]${}_{D}^{24}$ −0.301 (0.85, DMSO).

#### 4.1.9. Synthesis of 1-(3-bromobenzyl)-2-(hydroxymethyl) Piperidine-3,4,5-triol (**12i**)

Compound **12i** was prepared in a manner similar to the procedure described for the preparation of **12a** to yield a solid. The residue was purified using silica gel column chromatography (dichloromethane: methyl alcohol = 15:1) to produce a yellow solid (80%). ^1^H NMR (400 MHz, DMSO-*d*_6_) δ 7.50 (d, *J* = 7.8 Hz, 2H), 7.29 (d, *J* = 8.0 Hz, 2H), 4.75 (d, *J* = 22.5 Hz, 2H), 4.61 (s, 1H), 4.40 (s, 1H), 4.21 (d, *J* = 13.7 Hz, 1H), 3.94 (d, *J* = 10.7 Hz, 1H), 3.64 (s, 1H), 3.11 (d, *J* = 13.8 Hz, 3H), 2.94 (s, 1H), 2.57 (d, *J* = 10.9 Hz, 1H), 2.02 (s, 1H), 1.74 (d, *J* = 14.1 Hz, 1H); ^13^C NMR (100 MHz, DMSO-*d*_6_) δ 142.71, 131.21, 130.21, 129.51, 127.78, 121.58, 79.03, 70.81, 69.10, 67.81, 59.69, 56.78, 55.72; HRMS: C_13_H_18_BrNO_4_ for +, calculated 331.0414, found 331.0493; [α]${}_{D}^{24}$ −0.182 (0.62, DMSO).

#### 4.1.10. Synthesis of 2-(hydroxymethyl)-1-(3-iodobenzyl) Piperidine-3,4,5-triol (**12j**)

Compound **12j** was prepared in a manner similar to the procedure described for the preparation of **12a** to yield a solid. The residue was purified using silica gel column chromatography (dichloromethane: methyl alcohol = 15:1) to produce a yellow solid (91%). ^1^H NMR (400 MHz, DMSO-*d*_6_) δ 7.67 (d, *J* = 7.9 Hz, 2H), 7.14 (d, *J* = 7.9 Hz, 2H), 4.77 (d, *J* = 5.3 Hz, 1H), 4.71 (d, *J* = 4.2 Hz, 1H), 4.60 (d, *J* = 4.5 Hz, 1H), 4.39 (s, 1H), 4.20 (d, *J* = 14.0 Hz, 1H), 3.93 (d, *J* = 11.9 Hz, 1H), 3.66–3.58 (m, 1H), 3.16–3.03 (m, 3H), 2.97–2.89 (m, 1H), 2.56 (dd, *J* = 10.9, 4.2 Hz, 1H), 2.00 (d, *J* = 7.5 Hz, 1H), 1.71 (t, *J* = 10.7 Hz, 1H); ^13^C NMR (100 MHz, DMSO-*d*_6_) δ 217.68, 142.65, 137.10, 135.35, 130.26, 128.21, 94.69, 79.06, 70.87, 69.12, 67.87, 59.75, 56.80, 55.66, 40.37, 39.77; HRMS: C_13_H_18_INO_4_ for +, calculated 379.0276, found 379.0350; [α]${}_{D}^{20}$ −0.285 (0.75, DMSO).

#### 4.1.11. Synthesis of 2-(hydroxymethyl)-1-(3-methylbenzyl) Piperidine-3,4,5-triol (**12k**)

Compound **12k** was prepared in a manner similar to the procedure described for the preparation of **12a** to yield a solid. The residue was purified using silica gel column chromatography (dichloromethane: methyl alcohol = 10:1) to produce a white solid (90%). ^1^H NMR (400 MHz, DMSO-*d*_6_) δ 7.19 (d, *J* = 7.6 Hz, 2H), 7.11 (d, *J* = 7.6 Hz, 2H), 4.75 (d, *J* = 5.2 Hz, 1H), 4.69 (d, *J* = 4.1 Hz, 1H), 4.57 (d, *J* = 4.5 Hz, 1H), 4.32 (t, *J* = 4.8 Hz, 1H), 4.19 (d, *J* = 13.4 Hz, 1H), 3.95 (d, *J* = 10.2 Hz, 1H), 3.69–3.60 (m, 1H), 3.15–3.01 (m, 3H), 2.95–2.88 (m, 1H), 2.61 (dd, *J* = 11.0, 4.5 Hz, 1H), 2.51 (s, 1H), 1.97 (d, *J* = 8.7 Hz, 1H), 1.68 (t, *J* = 10.7 Hz, 1H); ^13^C NMR (100 MHz, DMSO-*d*_6_) δ 139.40, 137.04, 129.37, 127.94, 127.29, 125.93, 79.12, 70.92, 69.14, 68.01, 59.76, 56.65; HRMS: C_14_H_21_NO_4_ for +, calculated 267.1466, found 267.1527; [α]${}_{D}^{24}$ −0.151 (0.53, DMSO).

#### 4.1.12. Synthesis of 2-(hydroxymethyl)-1-(3-methoxybenzyl) Piperidine-3,4,5-triol (**12l**)

Compound **12l** was prepared in a manner similar to the procedure described for the preparation of **12a** to yield a solid. The residue was purified using silica gel column chromatography (dichloromethane: methyl alcohol = 10:1) to produce a white solid (80%). ^1^H NMR (400 MHz, DMSO-*d*_6_) δ4.75 (d, *J* = 5.2 Hz, 1H), 4.69 (d, *J* = 4.1 Hz, 1H), 4.57 (d, *J* = 4.5 Hz, 1H), 4.32 (t, *J* = 4.8 Hz, 1H), 4.19 (d, *J* = 13.4 Hz, 1H), 3.95 (d, *J* = 10.2 Hz, 1H), 3.69–3.60 (m, 1H), 3.15–3.01 (m, 3H), 2.95–2.88 (m, 1H), 2.61 (dd, *J* = 11.0, 4.5 Hz, 1H), 2.51 (s, 1H), 1.97 (d, *J* = 8.7 Hz, 1H), 1.68 (t, *J* = 10.7 Hz, 1H); ^13^C NMR (100 MHz, DMSO-*d*_6_) δ 159.21, 129.23, 67.80, 62.78, 55.67, 54.90, 52.18, 7.28; HRMS: C_14_H_21_NO_5_ for +, calculated 283.1415, found 283.1492; [α]${}_{D}^{25}$ −0.145 (0.50, DMSO).

#### 4.1.13. Synthesis of 4-(benzyloxy)-3-methoxybenzaldehyde (**13**)

Benzyl chloride (36 mmol) was slowly added to a solution of compound **6** (30 mmol), K_2_CO_3_ (36 mmol) and 4-fluorobenzyl bromide (2.4 mmol) in ethyl alcohol. The mixture was refluxed overnight until compound **6** was depleted, as determined by TLC analysis. Then, the mixture was cooled to r.t. After filtration, the residue was purified using silica gel column chromatography (petroleum ether: ethyl acetate = 5:1) to produce a faint yellow solid at 90% yield.

#### 4.1.14. Synthesis of 4-(benzyloxy)-3-bromo-5-methoxybenzaldehyde (**14**)

Compound **14** was prepared in a manner similar to the procedure described for the preparation of **13** to yield a solid. The residue was purified using silica gel column chromatography (petroleum ether: ethyl acetate = 5:1) to produce a faint yellow solid at 88% yield. ^1^H NMR (400 MHz, CDCl_3_) δ 9.87 (s, 1H), 7.68 (s, 1H), 7.55 (d, *J* = 7.0 Hz, 2H), 7.44–7.33 (m, 4H), 5.18 (s, 2H), 3.97 (s, 3H).

#### 4.1.15. General Procedure for the Preparation of Alcohol Derivatives (**15a**–**15e**)

Sodium borohydride was added to a solution of vanillic derivatives (**8**, **9**, **10**, **13**, **14**) (8.96 mmol) in DMF at 0 °C. The mixture was stirred until compound **13** was exhausted. The residue was washed three times with EtOAc (100 mL) and H_2_O (100 mL), and then, the organic layer was combined. After evaporating under vacuum, the residue was purified using silica gel column chromatography (petroleum ether: ethyl acetate = 1:1) to produce alcohol derivatives **15a**–**15e** that were a faint yellow solid. ^1^H NMR for 15a (400 MHz, CDCl_3_) δ 7.46 (d, *J* = 7.2 Hz, 2H), 7.39 (t, *J* = 7.2 Hz, 2H), 7.33 (d, *J* = 7.0 Hz, 1H), 6.98 (s, 1H), 6.86 (q, *J* = 8.1 Hz, 2H), 5.19 (s, 2H), 4.64 (d, *J* = 5.6 Hz, 2H), 3.93 (s, 3H); ^1^H NMR for 15b (400 MHz, CDCl_3_) δ 9.87 (s, 1H), 7.68 (s, 1H), 7.55 (d, *J* = 7.0 Hz, 2H), 7.44–7.33 (m, 4H), 5.18 (s, 2H), 3.97 (s, 3H).

#### 4.1.16. General Procedure for the Preparation of Benzyl Bromide Derivatives (**16a**–**16e**)

A solution of phosphorus tribromide in DCM was slowly added to a solution of alcohol derivatives **15a**–**15e** in DCM at 0 °C. The mixture was stirred at 0 °C until the compound was exhausted, as assessed using TLC. The residue was washed three times with H_2_O and saturated aq NaHCO_3_, dried over anhydrous Na_2_SO_4_, and then evaporated under vacuum to obtain the benzyl bromide derivatives **16a**–**16e**. The residues were used as crude compounds without purification.

#### 4.1.17. Synthesis of 2-(hydroxymethyl)-1-(3-methoxy-4-phenoxybenzyl) Piperidine-3,4,5-triol (**17a**)

Compound **17a** was prepared in a manner similar to the procedure described for the preparation of **12a** to yield a solid. The residue was purified using silica gel column chromatography (dichloromethane: methyl alcohol = 10:1) to produce a pink solid at 78% yield. ^1^H NMR (400 MHz, DMSO-*d*_6_) δ 7.38 (d, *J* = 7.0 Hz, 2H), 7.32 (t, *J* = 7.2 Hz, 2H), 7.29–7.21 (m, 1H), 6.89 (d, *J* = 9.2 Hz, 2H), 6.72 (d, *J* = 7.6 Hz, 1H), 4.97 (s, 2H), 4.57 (s, 1H), 4.31 (s, 1H), 4.09 (d, *J* = 13.2 Hz, 1H), 3.87 (d, *J* = 9.0 Hz, 1H), 3.68 (s, 3H), 3.58 (d, *J* = 5.0 Hz, 1H), 3.11–2.91 (m, 3H), 2.84 (d, *J* = 16.1 Hz, 2H), 2.66 (s, 1H), 2.57 (d, *J* = 7.0 Hz, 1H), 1.89 (d, J = 7.1 Hz, 1H), 1.60 (t, *J* = 10.5 Hz, 1H); ^13^C NMR (100 MHz, DMSO-*d*_6_) δ 162.33, 153.12, 144.24, 137.38, 123.80, 116.28, 112.82, 79.01, 70.83, 69.12, 67.83, 59.83, 56.71, 56.01, 55.80; HRMS: C_21_H_27_NO_6_ for +, calculated 389.1833, found 389.1907; [α]${}_{D}^{21}$ −0.206 (0.81, DMSO).

#### 4.1.18. Synthesis of 1-(3-bromo-5-methoxy-4-phenoxybenzyl)-2-(hydroxymethyl) Piperidizne-3,4,5-triol (**17b**)

Compound **17b** was prepared in a similar manner to the procedure described for the preparation of **12a** to yield a solid. The residue was purified using silica gel column chromatography (dichloromethane: methyl alcohol = 10:1) to produce a pink solid at 70% yield. ^1^H NMR (400 MHz, DMSO-*d*_6_) δ 7.35 (dd, *J* = 14.3, 7.5 Hz, 1H), 7.16 (d, *J* = 6.0 Hz, 2H), 7.05 (t, *J* = 8.6 Hz, 1H), 4.78 (d, *J* = 5.3 Hz, 1H), 4.72 (d, *J* = 4.0 Hz, 1H), 4.62 (d, *J* = 4.3 Hz, 1H), 4.41 (s, 1H), 4.26 (d, *J* = 14.0 Hz, 1H), 3.94 (d, *J* = 11.6 Hz, 1H), 3.67–3.59 (m, 1H), 3.20–3.05 (m, 3H), 2.98–2.90 (m, 1H), 2.60 (dd, *J* = 11.1, 4.5 Hz, 1H), 2.03 (d, *J* = 8.4 Hz, 1H), 1.75 (t, *J* = 10.8 Hz, 1H); ^13^C NMR (100 MHz, DMSO-*d*_6_) δ 162.33, 153.12, 144.24, 137.38, 123.80, 116.28, 112.82, 79.01, 70.83, 69.12, 67.83, 59.83, 56.71, 56.01, 55.80; HRMS: C_21_H_26_BrNO_6_ for +, calculated 467.0939, found 467.1037; [α]${}_{D}^{23}$ −0.118 (0.62, DMSO).

#### 4.1.19. Synthesis of 1-(3-bromo-4,5-dimethoxybenzyl)-2-(hydroxymethyl) Piperidine-3,4,5-triol (**17c**)

Compound **17c** was prepared in a manner similar to the procedure described for the preparation of **12a** to yield a solid. The residue was purified using silica gel column chromatography (dichloromethane: methyl alcohol = 5:1) to produce a pink solid at 66% yield. ^1^H NMR (400 MHz, DMSO-*d*_6_) δ 7.11 (s, 1H), 7.00 (s, 1H), 4.78 (d, *J* = 5.3 Hz, 1H), 4.72 (d, *J* = 4.0 Hz, 1H), 4.63 (d, *J* = 4.3 Hz, 1H), 4.41 (s, 1H), 4.16 (d, *J* = 13.8 Hz, 1H), 3.91 (d, *J* = 11.1 Hz, 1H), 3.79 (s, 3H), 3.69 (s, 3H), 3.64–3.55 (m, 1H), 3.18–3.10 (m, 1H), 3.06 (d, *J* = 13.2 Hz, 2H), 2.92 (td, *J* = 8.9, 4.4 Hz, 1H), 2.59 (dd, *J* = 11.0, 4.5 Hz, 1H), 1.98 (d, *J* = 9.7 Hz, 1H), 1.71 (t, *J* = 10.7 Hz, 1H); ^13^C NMR (100 MHz, DMSO-*d*_6_) δ 162.33, 153.12, 144.24, 137.38, 123.80, 116.28, 112.82, 79.01, 70.83, 69.12, 67.83, 59.83, 56.71, 56.01, 55.80; HRMS: C_15_H_22_BrNO_6_ for +, calculated 391.0626, found 391.0696, [α]${}_{D}^{21}$ −0.186 (0.50, DMSO).

#### 4.1.20. Synthesis of 1-(2,3-dibromo-4,5-dimethoxybenzyl)-2-(hydroxymethyl) Piperidine-3, 4, 5-triol (**17d**)

Compound **17d** was prepared in a manner similar to the procedure described for the preparation of **12a** to yield a solid. The residue was purified using silica gel column chromatography (dichloromethane: methyl alcohol = 5:1) to produce a pink solid at 50% yield. ^1^H NMR (400 MHz, DMSO-*d*_6_) δ 7.33 (s, 1H), 4.83 (d, *J* = 5.4 Hz, 1H), 4.78 (d, *J* = 4.2 Hz, 1H), 4.67 (d, *J* = 4.5 Hz, 1H), 4.45 (t, *J* = 4.9 Hz, 1H), 4.27 (d, *J* = 15.2 Hz, 1H), 3.93–3.85 (m, 1H), 3.81 (s, 3H), 3.71 (s, 3H), 3.59–3.51 (m, 1H), 3.20 (ddd, *J* = 14.3, 9.4, 4.5 Hz, 1H), 3.10–3.02 (m, 1H), 2.98 (td, *J* = 8.6, 4.2 Hz, 1H), 2.57 (dd, *J* = 11.2, 4.6 Hz, 1H), 2.14 (dd, *J* = 8.7, 3.7 Hz, 1H), 1.89 (t, *J* = 10.8 Hz, 1H); ^13^C NMR (100 MHz, DMSO-*d*_6_) δ 152.74, 146.15, 137.66, 120.90, 115.89, 114.12, 79.30, 71.63, 69.72, 68.82, 60.82, 60.51, 57.72, 56.57; HRMS: C_15_H_21_Br_2_NO_6_ for +, calculated 468.9731, found 470.9769; [α]${}_{D}^{22}$ −0.277 (1.18, DMSO).

#### 4.1.21. Synthesis of 2-(hydroxymethyl)-1-(2,3,6-tribromo-4,5-dimethoxybenzyl)-2-piperidine-3, 4, 5-triol (**17e**)

Compound **17e** was prepared in a manner similar to the procedure described for the preparation of **12a** to yield a solid. The residue was purified using silica gel column chromatography (dichloromethane: methyl alcohol = 3:1) to produce a pink solid at 49% yield. ^1^H NMR (400 MHz, DMSO-*d*_6_) δ 4.89 (d, *J* = 5.1 Hz, 1H), 4.76 (s, 1H), 4.64 (d, *J* = 3.6 Hz, 1H), 4.54–4.47 (m, 2H), 4.01 (d, *J* = 11.3 Hz, 1H), 3.85 (s, 3H), 3.82 (s, 3H), 3.78 (d, *J* = 12.6 Hz, 1H), 3.01–2.90 (m, 2H), 2.89 (s, 2H), 2.73 (s, 1H), 2.37 (d, *J* = 10.7 Hz, 1H), 2.15–2.07 (m, 1H), 1.97 (t, *J* = 10.1 Hz, 1H); ^13^C NMR (100 MHz, DMSO-*d*_6_) δ 150.74, 150.18, 134.97, 123.61, 122.22, 121.38, 78.80, 71.34, 70.31, 69.51, 61.35, 58.97, 55.48; HRMS: C_15_H_20_Br_3_NO_6_ for +, calculated 546.8836, found 548.8870; [α]${}_{D}^{22}$ −0.097 (0.57, DMSO).

#### 4.1.22. Synthesis of 1-(4-hydroxy-3-methoxybenzyl)-2-(hydroxymethyl) piperidine-3,4,5-triol (**18a**)

Aqueous 2 M HCl was added to a cooled solution of **17a** (800 mg) in ethanol/EtOAc (*v/v* = 1:1), adjusting the pH to 5–8. Oxygen was purged by flushing the mixture with nitrogen for 15 min, and palladium on carbon (80 mg) was added to the solution. Hydrogen was bubbled through the vigorously stirred reaction mixture until compound **17a** was exhausted, as assessed using TLC analysis. The residue was evaporated under vacuum and purified using silica gel column chromatography (petroleum ether: ethyl acetate = 1:1) to produce a white solid at 67% yield. ^1^H NMR (400 MHz, DMSO-*d*_6_) δ 8.79 (s, 1H), 6.86 (s, 1H), 6.69 (s, 2H), 4.83–4.70 (m, 2H), 4.63 (d, *J* = 3.7 Hz, 1H), 4.34 (s, 1H), 4.10 (d, *J* = 13.2 Hz, 1H), 3.93 (d, *J* = 11.1 Hz, 1H), 3.68–3.59 (m, 1H), 3.42 (s, 1H), 3.18–3.04 (m, 2H), 2.99 (d, *J* = 13.2 Hz, 1H), 2.95–2.87 (m, 1H), 2.65 (dd, *J* = 10.9, 4.1 Hz, 1H), 1.95 (d, *J* = 8.8 Hz, 1H), 1.66 (t, *J* = 10.7 Hz, 1H).^13^C NMR (100 MHz, DMSO-*d*_6_) δ 147.78 (s), 145.73 (s), 130.55 (s), 121.80 (s), 115.48 (s), 113.48 (s), 79.66 (s), 71.43 (s), 69.73 (s), 68.42 (s), 60.14 (s), 57.06 (s), 56.81 (s), 56.07 (s).HRMS: C_14_H_21_NO_6_ for +, calculated 299.1364, found 299.1455; [α]${}_{24}^{D}$ 0.011 (0.82, DMSO).

#### 4.1.23. Synthesis of 1-(3-bromo-4-hydroxy-5-methoxybenzyl)-2-(hydroxymethyl) piperidine-3,4,5-triol (**18b**)

Compound **18b** was prepared in a manner similar to the procedure described for the preparation of **18a** to yield a solid (50%). ^1^H NMR (400 MHz, DMSO-*d*_6_) δ 7.01 (s, 1H), 6.90 (s, 1H), 4.99–4.19 (m, 5H), 4.11 (d, *J* = 13.3 Hz, 1H), 3.92 (d, *J* = 11.4 Hz, 1H), 3.79 (s, 3H), 3.65 (d, *J* = 8.8 Hz, 1H), 3.14–3.08 (m, 1H), 3.04 (d, *J* = 13.3 Hz, 1H), 2.95 (t, *J* = 8.7 Hz, 1H), 2.65 (d, *J* = 6.8 Hz, 1H), 1.99 (d, *J* = 7.9 Hz, 1H), 1.71 (t, *J* = 10.6 Hz, 1H).^13^C NMR (100 MHz, DMSO-*d*_6_) δ 148.58 (s), 142.93 (s), 131.54 (s), 124.51 (s), 112.10 (s), 109.34 (s), 79.41 (s), 71.20 (s), 69.53 (s), 68.13 (s), 59.94 (s), 56.91 (s), 56.43 (s), 56.01 (s). HRMS: C_14_H_20_BrNO_6_ for +, calculated 377.0469, found 377.0544; [α]${}_{D}^{21}$ −0.021 (0.58, DMSO).

### 4.2. X-Ray Diffraction Experiment

A colourless single crystal with dimensions of 0.160 × 0.160 × 0.140 mm^3^ was selected and mounted on a glass fibre. Reflection data were collected at room temperature on a Bruker SMART APEX II area detector diffractometer [31] equipped with a graphite-monochromatic Mo Kα radiation (λ = 0.071073 nm) at 296(2) K with ω-2θ scan mode. Empirical adsorption corrections were applied to all data using SADABS. The structures were solved by direct methods and refined by full-matrix least squares on F2 using SHELXTL 97 software [32].

**Figure 3:**
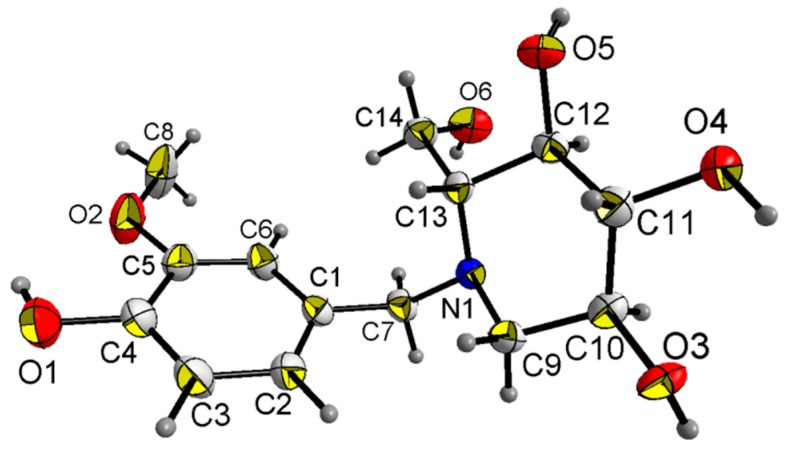
Crystal structure of compound **18a** with 50% thermal ellipsoids.

**Figure 4:**
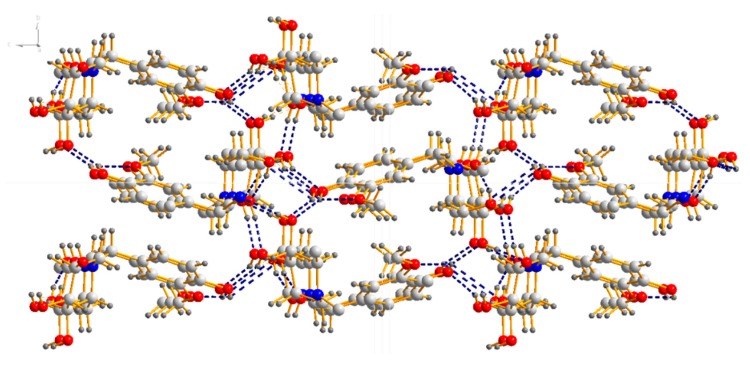
Three-dimensional hydrogen bond network structure of **18a**.

X-ray diffraction analysis shows that compound **18a** crystallizes in the orthorhombic space group P212121; the crystal data along with the collection parameters are shown in Table 3. Table 4 shows selected bond lengths and bond angles. In the molecule of compound **18a** (Figure 3), bond lengths and angles within synthesis of 1-(4-hydroxy-3-methoxybenzyl)-2-(hydroxymethyl) piperidine-3,4,5-triol are very similar to those given in the literature for N-t-Butoxycarbonyl-1-deoxynojiri,mycin [33]. The six-membered ring adopts chair conformations. The three hydroxyl groups and one hydroxyl methyl group at the six-membered ring are all in equatorial positions, while those groups at six-membered ring are all axially oriented in the literature [33]. There is also a substituted benzyl group attached to the nitrogen atom(N1) at the six-membered ring. The C1–C7–N1–C9 and C1–C7–N1–C13 torsion angles are 73.95° and 52.255°, respectively. All the non-hydrogen atoms in the substituted benzyl group are approximately in the same plane. The dihedral angle between the plane of the six-membered ring and the C1-C6 phenyl ring is 70.498°. The molecule shows abundant intramolecular O–H…O hydrogen bonds and O–H…N hydrogen bond, and those intramolecular hydrogen bonds link adjacent molecules to form a three-dimensional hydrogen bond network structure (Figure 4).

All non-hydrogen atoms were located by direct methods and subsequent difference Fourier syntheses. The hydrogen atoms bound to carbon were located by geometrical calculations, and their positions and thermal parameters were fixed during the structure refinement. Crystallographic date and pertinent information are given in Table 3, selected bond lengths and angles in Table 4 and geometric parameters of hydrogen bonds in Table 5. 

### 4.3. In Vitro Assay of α-Glucosidase Inhibitory Activity

In vitro assays of α-glucosidase (EC number: 3.2.1.20, Saccharomyces cerevisiae, purchased from Sigma-Aldrich, Saint Louis, MI, USA) inhibitory activity were carried out in 96-well microplates according to the method of Zhu et al with slight modifications [34]. The inhibitors were dissolved in a mixture of DMSO and 0.1 M phosphate buffer, pH 6.8 (*v/v* = 1:4). Ten microliters of inhibitor solution, 10 μL of 0.5 U/mL α-glucosidase, and 1.5 μL of 0.1 M beta-mercaptoethanol were mixed with 168.5 μL 0.1 M phosphate buffer, and then, the mixture was pre-incubated for 20 min at 37 °C. Twenty microliters of 1 mM PNPG was added to the mixture, and the solution was incubated for another 5 min at 37 °C. After incubation, the absorbance of the mixture was measured at 405 nm using a microplate reader (SpectraMax M2). Acarbose was used as a positive control. All experiments were performed in triplicate. The inhibition percentage was calculated in accordance with the following formula:

Inhibition (%) = (1 − As/Ac) × 100,

where As is the absorbance of the tested sample and Ac is the absorbance of the control without inhibitor. The IC_50_ value was defined as a concentration of sample inhibiting 50% of α-glucosidase activity under the previously mentioned conditions and was calculated using Origin 8.4 by applying logarithmic regression analysis.

The same protocol was followed for the experiments reported in Figure 1 except that the inhibitor concentration was 0.25 mM and the substrate concentration was varied as indicated (0.1, 0.2, 0.8, 1.0, 1.2, 1.6, 1.8, 2.0, 2.5, 5 mM). The Km and Vmax values were calculated using Origin 8.4, and then, the K*_i_* value was calculated using the formula that was mentioned in Section 2.3.

### 4.4. Molecular Docking Simulation

The three-dimensional structure of α-glucosidase is not accessible from the Protein Data Bank. However, oligo-1,6-glucosidase shares 72% identity and 85% similarity with α-glucosidase sequence. Therefore, we chose the three-dimensional structure of oligo-1,6-glucosidase (PDB: 3A4A) as our receptor proteins. The three-dimensional structure of oligo-1,6-glucosidase was downloaded from the Protein Data Bank. All water molecules were removed with using AutoDock Tools. Polar hydrogens were added to the protein structure. Here, the bound ligand area was utilized to generate a grid box for oligo-1,6-glucosidase with binding site region as center × 21.544, center y −7.476, center z −24.158 with size as 40, 40, and 40, respectively. Ligand were built and energy minimized (the convergence conditions were set as 0.01 Kcal/mol) using Sybyl 2.0. Lamarckian genetic algorithm in Autodock was applied to calculate the possible conformation of the ligand that bound to yeast glucoamylase. In the case of compound **18a**, calculations were performed using the X-ray coordinate as the input structure. The top-ranked docked conformations of the inhibitors were selected to illustrate the interactions between α-glucosidase and the inhibitors. The binding mode, the binding site, and the binding energy were analysed in detail to interpret the inhibitory mechanism.

## Figures and Tables

**Table 1:**
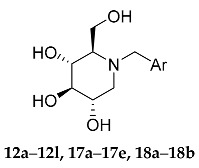
The inhibition activities of N-benzyl 1-DNJ derivatives towards α-glucosidase.

Compound	Ar	IC_50_ (mM) ± SEM	Compound	Ar	IC_50_ (mM) ± SEM
**12a**	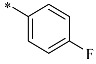	9.8 ± 0.31	**12k**	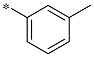	10.3 ± 0.21
**12b**	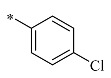	11.22 ± 0.02	**12l**	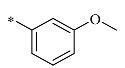	1.253 ± 0.34
**12c**	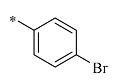	12.55 ± 0.03	**17a**	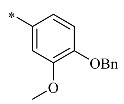	15.33 ± 0.02
**12d**	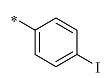	9.88 ± 0.05	**17b**	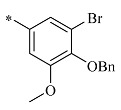	15.68 ± 0.06
**12e**	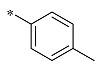	8.65 ± 0.08	**17c**	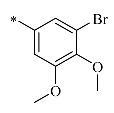	1.056 ± 0.11
**12f**	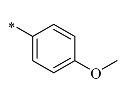	1.017 ± 0.23	**17d**	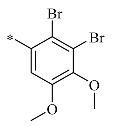	1.142 ± 0.09
**12g**	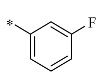	10.23 ± 0.02	**17e**	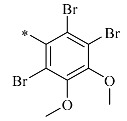	9.57 ± 0.08
**12h**	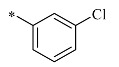	15.32 ± 0.04	**18a**	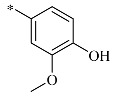	0.207 ± 0.11
**12i**	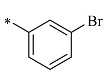	15.66 ± 0.04	**18b**	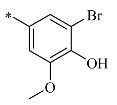	0.276 ± 0.13
**12j**	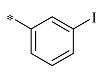	10.54 ± 0.04	Acarbose	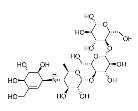	0.353 ± 0.09

**Table 2:** Inhibitory kinetic parameter estimation results for acarbose and **18a** towards α-glucosidase.

	Vmax/*10^−6^ mmol/min	*K*m/mM	*K_i_*/mM	IC_50_/mM
blank group	4.68 ± 0.40	2.15 ± 0.43		
acarbose	4.40 ± 0.42		0.113 ± 0.45	0.353 ± 0.09
18a	3.56 ± 0.28		0.110 ± 0.30	0.207 ± 0.11

**Table 3:** Crystal and structure refinement date for **18a**.

**Empirical formula**	C14 H21NO6	**Z**	4
**Formula weight**	299.32	**Dc (g cm^−3^)**	1.380
**Crystal system**	Orthorhombic	**Mu (MoKa) (/mm)**	0.108
**Space group**	P212121	**F (000)**	640
**a(Å)**	7.760(2)	**Crystal size (mm^3^)**	0.160 × 0.160 × 0.140
**b(Å)**	9.028(2)	**Θ range for date collection (°)**	2.464 to 25.492
**c(Å)**	20.563(6)	**Reflections collected**	11,171
**α(°)**	90.00	**Independent reflection**	2679 [R(int) = 0.0249]
**β(°)**	90.00	**Goodness-of-fit on F2**	1.039
**γ(°)**	90.00	**Final R indices [I > 2σ(I)] a**	R1 = 0.0352, wR2 = 0.0873
**V(Å 3)**	1440.6(7)	**R indices (all date) a**	R1 = 0.0386, wR2 = 0.0896

^a^*R*_1_ = Σ ║*F_o_*│ − │*F_c_*║/Σ│*F_o_*│; *wR*_2_ = [*Σw*(*F_o_*^2^ − *F_c_*^2^)_2_/*Σw*(*F_o_*^2^)^2^]^1/2^.

**Table 4:** Selected bond lengths (Å) and bond angles (°).

**Bond**	**Dist.**	**Bond**	**Dist.**	**Bond**	**Dist.**
C (1)-C (2)	1.381(4)	C (1)-C (6)	1.396(4)	C (1)-C (7)	1.522(3)
C (2)-C (3)	1.389(4)	C(3)-C(4)	1.380(4)	C (4)-O (1)	1.361(3)
C (4)-C (5)	1.398(4)	C(5)-O(2)	1.370(3)	C (5)-C (6)	1.385(4)
C (7)-N (1)	1.487(3)	C(8)-O(2)	1.406(4)	C(9)-N(1)	1.468(3)
C (9)-C (10)	1.512(3)	C(10)-O(3)	1.426(3)	C(10)-C(11)	1.506(4)
C (11)-O (4)	1.438(3)	C(11)-C(12)	1.507(3)	C(12)-O(5)	1.425(3)
C(12)-C(13)	1.535(3)	C(13)-N(1)	1.478(3)	C(13)-C(14)	1.521(3)
C(14)-O(6)	1.416(3)				
**Angle**	**(** **°** **)**	**Angle**	**(** **°** **)**	**Angle**	**(** **°** **)**
C(2)-C(1)-C(6)	117.8(2)	C(2)-C(1)-C(7)	123.2(2)	C(6)-C(1)-C(7)	118.9(2)
C(1)-C(2)-C(3)	121.2(3)	C(4)-C(3)-C(2)	120.7(3)	O(1)-C(4)-C(3)	118.8(3)
O(1)-C(4)-C(5)	122.4(3)	C(3)-C(4)-C(5)	118.9(2)	O(2)-C(5)-C(6)	125.2(3)
O(2)-C(5)-C(4)	114.9(2)	C(6)-C(5)-C(4)	119.9(2)	C(5)-C(6)-C(1)	121.4(3)
N(1)-C(7)-C(1)	118.1(2)	N(1)-C(9)-C(10)	110.56(19)	O(3)-C(10)-C(11)	106.66(19)
O(3)-C(10)-C(9)	110.8(2)	C(11)-C(10)-C(9)	111.5(2)	O(4)-C(11)-C(10)	110.3(2)
O(4)-C(11)-C(12)	107.5(2)	C(10)-C(11)-C(12)	112.82(19)	O(5)-C(12)-C(11)	108.51(19)
O(5)-C(12)-C(13)	109.10(19)	C(11)-C(12)-C(13)	112.8(2)	N(1)-C(13)-C(14)	114.0(2)
N(1)-C(13)-C(12)	108.88(18)	C(14)-C(13)-C(12)	108.42(19)	O(6)-C(14)-C(13)	112.0(2)
C(9)-N(1)-C(13)	109.12(19)	C(9)-N(1)-C(7)	112.42(19)	C(13)-N(1)-C(7)	115.62(19)
C(5)-O(2)-C(8)	117.8(2)				

**Table 5:** Hydrogen bond lengths (Å) and bond angles (°) of **18a**.

18a				
O(6)–H(7)⋅⋅⋅O(3) #1	0.82	2.04	2.828(3)	161.4
O(5)–H(5)⋅⋅⋅N(1)	0.82	2.11	2.923(3)	171.0
O(4)–H(4)⋅⋅⋅O(6) #2	0.082	2.64	3.096(3)	116.6
O(4)–H(4)⋅⋅⋅O(1) #3	0.82	2.17	2.928(3)	154.6
O(3)–H(3)⋅⋅⋅O(4) #4	0.82	1.86	2.670(3)	169.5
O(1)–H(1)⋅⋅⋅O(5) #5	0.82	2.06	2.784(3)	146.5
O(1)–H(1)⋅⋅⋅O(2)	0.82	2.27	2.706(3)	114.0
C(10)–H(10)⋅⋅⋅O(6) #6	0.98	2.53	3.277(3)	133.4
C(7)–H(7B)⋅⋅⋅O(3) #1	0.97	2.60	3.566(3)	173.4

Symmetry codes: **1**, #1: x + 1, y, z; #2: x − 1, y, z; #3: −x − 1/2, −y + 1, z + 1/2; #4: −x − 1, y − 1/2, −z + 1/2; #5: x + 1/2, −y + 3/2, −z; #6: −x, y − 1/2, −z + 1/2.
